# P-2140. Clinical Review of Respiratory Tract Infections Caused by *Exophiala dermatitidis* Misidentified as genus *Rhodotorula*: Case Series

**DOI:** 10.1093/ofid/ofae631.2295

**Published:** 2025-01-29

**Authors:** Daichi Setoguchi, Naoki Iwanaga, Tatsuro Hirayama, Masataka Yoshida, Kazuaki Takeda, Shotaro Ide, Masato Tashiro, Takahiro Takazono, Kosuke Kosai, Koichi Izumikawa, Katsunori Yanagihara, Hiroshi Mukae

**Affiliations:** Nagasaki University, Nagasaki, Nagasaki, Japan; Nagasaki University Hospital, Nagasaki, Nagasaki, Japan; Nagasaki University, Nagasaki, Nagasaki, Japan; Nagasaki University Hospital, Nagasaki, Nagasaki, Japan; Nagasaki University Hospital, Nagasaki, Nagasaki, Japan; Nagasaki University Hospital, Nagasaki, Nagasaki, Japan; Nagasaki University Graduate School of Biomedical Sciences, Lomita, California; Nagasaki University Graduate School of Biomedical Sciences, Lomita, California; Nagasaki University, Nagasaki, Nagasaki, Japan; Nagasaki University, Nagasaki, Nagasaki, Japan; Nagasaki University, Nagasaki, Nagasaki, Japan; Nagasaki University, Nagasaki, Nagasaki, Japan

## Abstract

**Background:**

*Exophiala dermatitidis* is a black fungus isolated from wet environments such as soil and baths. Human infections have been reported chiefly as skin and soft tissue infections, and respiratory infections have been extremely rare. The fungus is dimorphic and known to be yeast-like in culture, changing to filamentous fungi over time. The fungus is often misidentified as yeast during the first few days of culture, and preliminary experiments have shown that it can be misidentified as *Rhodotorula* spp. by commercially available yeast identification kits.

**Methods:**

Genetic identification was performed using 44 strains isolated from airway specimens over the past 11 years from July 2012 to September 2023, except for one difficult to culture, by determining the sequence of the ITS and D1/D2 regions. Cases genetically identified with *E. dermatitidis* were examined retrospectively for clinical information except for seven cases identified of the same cases.

**Results:**

Of the 43 strains identified genetically, 22 were identified as *E. dermatitidis*, indicating that they were misidentified in approximately 50% of the cases; analysis of the clinical information of the 15 cases identified as *E. dermatitidis* and for which a retrospective search was possible showed that chronic lower respiratory tract infection or pneumonia due to *E. dermatitidis* was definite in six cases, active infection was possible in five cases and airway colonization only in four cases. Comorbidities included pulmonary non-tuberculosis mycobacterium (NTM) in five cases, chronic progressive pulmonary aspergillosis (CPPA) in three cases, and pulmonary nocardiosis in one case, suggesting concurrent chronic pulmonary infections due to other microbes. Steroid and immunosuppressive agent use was observed in ten cases. Five of the six definite cases responded to azoles, and one case resolved spontaneously with only a reduction of the immunosuppressive drugs. Drug sensitivity testing showed that all cases were resistant to Candin and sensitive to azoles and polyenes.

**Conclusion:**

*E. dermatitidis* is a dimorphic fungus that is time-consuming to culture and may have been underestimated previously, misidentified as *Rhodotorula* spp. In addition to NTM and CPPA, *E. dermatitidis* might be differentiated in chronic lung infections.

**Disclosures:**

Hiroshi Mukae, M.D., Ph.D., AstraZeneca: Lecture fees|Gilead Sciences: Lecture fees|GSK: Lecture fees|MSD: Advisor/Consultant|MSD: Lecture fees|Pfizer: Lecture fees|Shionogi & Co., Ltd.: Advisor/Consultant|Shionogi & Co., Ltd.: Grant/Research Support|Shionogi & Co., Ltd.: Lecture fees

